# Antimicrobial resistance in *Enterobacteriaceae* from healthy broilers in Egypt: emergence of colistin-resistant and extended-spectrum β-lactamase-producing *Escherichia coli*

**DOI:** 10.1186/s13099-018-0266-5

**Published:** 2018-09-19

**Authors:** Amira A. Moawad, Helmut Hotzel, Heinrich Neubauer, Ralf Ehricht, Stefan Monecke, Herbert Tomaso, Hafez M. Hafez, Uwe Roesler, Hosny El-Adawy

**Affiliations:** 1 Friedrich-Loeffler-Institut (Federal Research Institute for Animal Health), Institute of Bacterial Infections and Zoonoses, Naumburger Str. 96a, 07743 Jena, Germany; 2 Provincial Laboratory, Institute of Animal Health Research, Mansoura, Egypt; 3 Institute for Poultry Diseases, Free University Berlin, Königsweg 63, 14163 Berlin, Germany; 4 Alere Technologies GmbH, Löbstedter Str. 103-105, 07749 Jena, Germany; 5 InfectoGnostics Research Campus Jena e. V., Philosophenweg 7, 07743 Jena, Germany; 6 Institute for Medical Microbiology and Hygiene, Medical Faculty Carl Gustav Carus, Technische Universität Dresden, Fetscherstr. 74, 01307 Dresden, Germany; 7 Institute for Animal Hygiene and Environmental Health, Free University Berlin, Robert-von Ostertag-Str. 7-13, 14163 Berlin, Germany; 8 Faculty of Veterinary Medicine, Kafrelsheikh University, Kafr El-Sheikh, 33516 Egypt

**Keywords:** *Enterobacteriaceae*, Antibiotic resistance, DNA microarray, ESBL, Colistin, Broiler

## Abstract

**Background:**

Poultry remains one of the most important reservoir for zoonotic multidrug resistant pathogens. The global rise of antimicrobial resistance in Gram-negative bacteria is of reasonable concern and demands intensified surveillance.

**Methods:**

In 2016, 576 cloacal swabs were collected from 48 broiler farms located in five governorates in northern Egypt. Isolates of *Enterobacteriaceae* could be cultivated on different media and were identified by MALDI-TOF MS and PCR. *Escherichia coli* isolates were genotyped by DNA-microarray-based assays. The antimicrobial susceptibility to 14 antibiotics was determined and resistance-associated genes were detected. The VITEK-2 system was applied for phenotypical confirmation of extended-spectrum β-lactamase-producing isolates. The determination of colistin resistance was carried out phenotypically using E-test and genotypically using PCR for detection of the *mcr*-1 gene.

**Results:**

Out of 576 samples, 72 representatives of *Enterobacteriaceae* were isolated and identified as 63 *E. coli* (87.5%), 5 *Enterobacter cloacae* (6.9%), 2 *Klebsiella pneumoniae* (2.8%) and 2 *Citrobacter* spp. (2.8%). Seven out of 56 cultivated *E. coli* (12.5%) were confirmed as ESBL-producing *E. coli* and one isolate (1.8%) as ESBL/carbapenemase-producing *E. coli*. Five out of 63 *E. coli* isolates (7.9%) recovered from different poultry flocks were phenotypically resistant to colistin and harboured *mcr*-1 gene.

**Conclusions:**

This is the first study reporting colistin resistance and emergence of multidrug resistance in *Enterobacteriaceae* isolated from healthy broilers in the Nile Delta region, Egypt. Colistin-resistant *E. coli* in poultry is of public health significance. The global rise of ESBL- and carbapenemase-producing Gram-negative bacteria demands intensified surveillance. ESBL-producing *E. coli* in poultry farms in Egypt are of major concern that emphasizes the possibility of spread of such strains to humans. The results also reinforce the need to develop strategies and to implement specific control procedures to reduce the use of antibiotics.

## Background

Poultry and their products are considered the main vehicle for pathogenic bacteria such as *Salmonella* (*S.*) serovars, *Escherichia* (*E.*) *coli* and *Klebsiella* (*K.*) spp. that cause foodborne infections in humans [1–3].

The prevalence of highly antibiotic-resistant *E. coli* was recorded in poultry meat more frequently than in all other kinds of meat [4, 5].

Extended-spectrum β-lactamases (ESBLs) are plasmid-encoded enzymes found in Gram-negative bacteria especially in *Enterobacteriaceae* conferring resistance to first, second and third generation cephalosporins while they are inhibited by clavulanic acid [6–10].

ESBL-producing *Enterobacteriaceae* have emerged as pathogens in both poultry and humans [7, 11]. Many ESBL-producers are additionally multiresistant to non-β-lactam antibiotics, including fluoroquinolones, aminoglycosides, trimethoprim, tetracyclines, sulfonamides and chloramphenicols [12, 13]. Resistance to cephalosporins is mediated by ampicillin class C β-lactamases (AmpC β-lactamase) and encoded by *bla*_CMY_ genes [14, 15]. Carbapenems are still the drugs of choice to treat infections with ESBL-producing *Enterobacteriaceae* in humans [16] and their increasing use reinforces the probability of resistance development to carbapenems among *Enterobacteriaceae* [17–19]. The coexistence of multiple ESBL and carbapenemase genes as well as other antibiotic resistance determinants on mobile elements is of a major concern that might lead to the emergence of organisms with resistance to all antibiotics [6, 20, 21].

Most ESBLs encoding genes in bacteria of clinical interest are located on plasmids [22]. These plasmids may also carry genes encoding resistance to other drug classes including aminoglycosides and fluoroquinolones [23]. Transmission of ESBLs genes can occur either by emerging bacterial clones or by horizontal gene transfer. In the latter case, plasmids containing resistance genes, spread between bacteria [22]. Colistin recently gained attention as a last-resort antibiotic for treatment of infections caused by multidrug resistant Gram-negative bacteria. In veterinary practice, colistin is a drug of choice for the treatment of frequent digestive tract infections caused by *E*. *coli* in food-producing animals [24]. The irrational use of colistin in veterinary practice may be the main cause of the increasing rate of colistin resistance. Recently, the emergence of colistin resistance has caused great concern [25, 26] and resistance mediated by the plasmid-borne *mcr*-1 gene has been detected worldwide in *Enterobacteriaceae* [27].

In some countries, antimicrobials are used in the poultry industry for treatment of diseased animals, prevention of diseases and promotion of growth [28–30]. In Egypt, *E. coli* infections are considered as one of the most serious diseases leading to economic losses in poultry production [31].

Unfortunately, there are no legislations in Egypt regulating the use of antibiotics. Some of them such as tetracycline, quinolones and beta-lactams are still used for non-therapeutic uses [32]. This improper use of antimicrobials leads to rapid selection of multiresistant strains of *Enterobacteriaceae* in poultry and plays a key role in the spread of antibiotic-resistant bacteria along the food chain to humans [33–35].

In recent years, matrix-assisted laser desorption/ionization time-of-flight mass spectrometry (MALDI-TOF MS) has been applied as a wide-range technique for bacterial identification [36]. Microarray systems are well-established tools for rapid genotypic characterization of bacteria and identification of resistance and virulence-associated determinants [37]. The data can be obtained in a single experiment with the benefit of saving time and money [38–40]. The broth microdilution method proved to be an easy and reliable method for determination of the minimum inhibitory concentration (MIC) of antibiotics and can be used as an alternative technique to agar diffusion test [41–43].

The use of a rapid molecular assay as an alternative to phenotypic detection was proved to be a useful option for detection of antibiotic resistance to frequently applied antimicrobial agents in poultry production [43].

The objective of this study was to gain insight into the antimicrobial susceptibility of *Enterobacteriaceae*, especially *E. coli* originating from healthy broilers from different districts in northern Egypt and to understand its public health significance. In addition, the prevalence of ESBL/carbapenemase-producing *E. coli* and colistin resistance were investigated.

## Methods

### Isolation and characterization of bacterial strains

During 2016, 576 cloacal swabs were randomly collected from apparently healthy broilers housed in 48 farms located in five governorates, namely: Dakahlia, Kafr El-Sheikh, Damietta, Gharbiya and Sharkiya, in the Nile Delta region, Egypt. An overview about investigated poultry farms, the number of birds and the number of collected samples are given in Table 1. Sampling was carried out using sterile cotton swabs. The collected samples were transported at 4 °C to the laboratory for microbiological examination. The samples were enriched in Buffered Peptone Water at 37 °C for 24 h and then streaked on MacConkey Agar and Eosin Methylene Blue (EMB) Agar (Oxoid, Manchester, UK), followed by further incubation at 37 °C for 24 h. For identification of ESBL-producing *Enterobacteriaceae*, the enriched bacterial cultures were cultivated on Brilliance™ ESBL agar (Oxoid GmbH, Wesel, Germany) at 37 °C for 24 h.

**Table 1:** Overview about investigated poultry farms and the number of collected samples

	Governorate					Total
	Dakahlia	Sharkiya	Gharbiya	Damietta	Kafr El-Sheikh	
No. of farms	20	5	4	9	10	48
No. of birds	58,000	18,000	12,000	28,000	28,000	144,000
No. of samples	232	72	48	112	112	576

### Identification by MALDI-TOF MS

Isolates were identified using MALDI-TOF MS [44]. Interpretation of results was performed according to the manufacturer’s recommendation: score of ≥ 2.3 represented reliable species level identification; score 2.0–2.29, probable species level identification; score 1.7–1.9, probable genus level identification, and score ≤ 1.7 was considered an unreliable identification [45].

### DNA extraction and purification

Genomic DNA was extracted from bacterial cultures using High Pure PCR Template Purification Kit (Roche Diagnostics, Mannheim, Germany) according to the instructions of the manufacturer.

### Identification of *E. coli* isolates using PCR

The identified *E. coli* isolates were confirmed at species level using a specific PCR assay targeting 16S rRNA genes with primers ECO-1 (5′-GAC CTC GGT TTA GTT CAC AGA-3′) and ECO-2 (5′-CAC ACG CTG ACG CTG ACC A-3′) which geared from previous study by Seidavi et al. [46]. The PCR reaction was carried out with the following amplification conditions: An initial denaturation step at 96 °C for 60 s was followed by 35 cycles of denaturation (96 °C for 15 s), annealing (58 °C for 60 s) and extension (72 °C at 45 s) with a final extension at 72 °C for 60 s. PCR resulted in 585 bp amplicons. PCR products were analyzed on a 1.5% agarose gel, stained with ethidium bromide and visualized under UV light.

### Genoserotyping of *E. coli* isolates using microarray assay

The serotypes of *E. coli* isolates were determined using the *E. coli* SeroGenoTyping AS-1 Kit (Alere Technologies GmbH, Jena, Germany). Five microliters of extracted RNA-free DNA (with a concentration of at least 100 ng/μl) were biotin-labeled by a primer extension amplification using *E. coli* SeroGenoTyping AS-1 Kit according to manufacturer’s instructions. The procedures for multiplex labelling, hybridization and data analysis were carried out as described in a previous study [47].

### Phenotypic antibiotic susceptibility testing

The antibiotic susceptibility testing of all isolates was performed with the MICRONAUT system using commercial 96-well microtiter plates (Merlin, Bornheim, Germany) as recommended by the manufacturer. This system allowed the determination of minimum inhibitory concentrations (MICs) of 14 antimicrobial agents (Tables 2, 3) in serial dilutions of the antibiotics. Over-night grown bacteria were suspended in NaCl solution (0.9%) to obtain a turbidity corresponding to a McFarland standard of 0.5 (Dr. Lange, CADAS photometer 30, Berlin, Germany). One hundred microliters of the suspension were diluted with 10 ml of Mueller–Hinton broth (Oxoid GmbH) resulting in a concentration of approximately 10^6^–10^7^ colony forming units (cfu)/ml. In total, 100 µl of the inoculum were given in each well of the plate. After sealing the plates, they were incubated for 18 h to 24 h at 37 °C. Reading of plates was done with a photometer (Merlin) at a wavelength of 620 nm. An optical density of > 0.1 was interpreted as an indication of growth. MICs were interpreted with the advanced expert system MCN-6 (Merlin) using the guidelines of the German Institute for Standardization (Deutsches Institut für Normung, Berlin, Germany). *E. coli* ATCC 25922, *E. coli* ATCC 35218 and *K. pneumoniae* ATCC 700603 were used as quality controls.

**Table 2:** Antibiotic susceptibility of 56 *Escherichia coli* isolates from broilers using broth microdilution test

Antibiotic	Class	S	I	R	Resistance rate (%)
Penicillin (PEN)	β-Lactam	0	1	55	98.2
Erythromycin (ERY)	Macrolide	1	1	54	96.4
Rifampicin (RAM)	Ansamycin	1	1	54	96.4
Trimethoprim/sulfamethoxazole (T/S)	Diaminopyrimidine/sulfonamide	19	1	36	64.3
Streptomycin (STR)	Aminoglycoside	12	14	30	53.6
Tetracycline (TET)	Tetracycline	24	4	28	50.0
Ceftazidime (CAZ)	β-Lactam (cephalosporin)	24	9	23	41.1
Amoxicillin/clavulanic acid (AMC)	β-Lactam/β-lactamase inhibitor	29	12	15	26.8
Chloramphenicol (CMP)	Non-classified	41	2	13	23.2
Ciprofloxacin (CIP)	Fluoroquinolone	38	6	12	21.4
Gentamicin (GEN)	Aminoglycoside	38	7	11	19.6
Levofloxacin (LEV)	Fluoroquinolone	35	13	8	14.3
Amikacin (AMK)	Aminoglycoside	43	7	6	10.7
Imipenem (IMP)	β-Lactam (carbapenem)	48	7	1	1.8

*S* susceptible, *I* intermediate, *R* resistant

**Table 3:** Antibiotic susceptibility of 9 *Enterobacteriaceae* isolates other than *E. coli* from broilers using broth microdilution test

Antibiotic	*Enterobacter cloacae* (n = 5)			Resistance rate (%)	*Citrobacter* spp. (n = 2)			Resistance rate (%)	*Klebsiella pneumoniae* (n = 2)			Resistance rate (%)
	S	I	R		S	I	R		S	I	R	
Penicillin (PEN)	0	0	5	100	0	0	2	100	0	0	2	100
Erythromycin (ERY)	0	0	5	100	1	0	1	50.0	0	0	2	100
Rifampicin (RAM)	0	0	5	100	0	0	2	100	0	0	2	100
Trimethoprim/sulfamethoxazole (T/S)	2	0	3	60.0	0	1	1	50.0	0	0	2	100
Streptomycin (STR)	1	0	4	80.0	0	0	2	100	0	0	2	100
Tetracycline (TET)	4	0	1	20.0	1	0	1	50.0	0	0	2	100
Ceftazidime (CAZ)	3	1	1	20.0	0	0	2	100	0	0	2	100
Amoxicillin/clavulanic acid (AMC)	1	0	4	80.0	1	1	0	0.0	0	0	2	100
Chloramphenicol (CMP)	4	0	1	20.0	2	0	0	0.0	0	0	2	100
Ciprofloxacin (CIP)	4	0	1	20.0	2	0	0	0.0	0	0	2	100
Gentamicin (GEN)	4	0	1	20.0	2	0	0	0.0	0	0	2	100
Levofloxacin (LEV)	4	0	1	20.0	2	0	0	0.0	0	0	2	100
Amikacin (AMK)	5	0	0	0.0	1	0	1	50.0	2	0	0	0.0
Imipenem (IMP)	4	1	0	0.0	2	0	0	0.0	1	1	0	0.0

*S* susceptible, *I* intermediate, *R* resistant

### Vitek-2 system

All suspected ESBL isolates were subsequently confirmed using an automated microdilution system (VITEK-2, bioMérieux Deutschland GmbH, Nürtingen, Germany) according to the instructions of the manufacturer. For this study, the test card AST-N289 was used that included the following antibiotics: piperacillin (PIP), piperacillin/tazobactam (TZP), cefotaxime (CTX), ceftazidime (CAZ), cefepime (FEB), aztreonam (ATM), imipenem (IMP), meropenem (MEM), amikacin (AMK), gentamicin (GEN), tobramycin (TOP), ciprofloxacin (CIP), moxifloxacin (MXF), tigecycline (TGC), fosfomycin (FOS), colistin (CT) and trimethoprim/sulfamethoxazole (T/S).

### Detection of antibiotic resistance and virulence-associated genes of *E. coli* isolates by microarray analysis

Antimicrobial resistance (AMR) genotypes and other resistance genes were ascertained using the CarbDetect AS-2 Kit and *E. coli* PanType AS-2 Kit, respectively (Alere Technologies GmbH). The data were automatically summarized by the “result collector”, a software tool provided by Alere Technologies GmbH. An antibiotic resistance genotype was assigned to be a carrier of a group of genes which have been described to confer resistance to a family of antibiotics (e.g., the genotype “*bla*_CTX-M1/15_, *bla*_TEM_” conferring resistance to 3rd generation cephalosporins).

The detection of virulence-associated genes was performed using *E. coli* PanType AS-2 Kit (Alere Technologies GmbH). Twenty-eight different combinations of genes encoding virulence factors associated with adhesion, fimbriae production, secretion systems, SPATE (serine protease auto-transporters), toxins and miscellaneous genes were detected. The genes were detected and analysed by the “result collector”, a software tool provided by Alere Technologies GmbH.

### Determination of colistin resistance

All identified *E. coli* isolates were tested for presence of plasmid-mediated *mcr*-1 gene using PCR [27]. Briefly, a PCR with 25 µL reaction mixture using CLR5-F (5′-CGG TCA GTC CGT TTG TTC-3′) and CLR5-R (5′-CTT GGT CGG TCT GTA GGG-3′) was performed with the following amplification conditions: initial denaturing at 96 °C for 60 s was followed by 35 cycles of denaturing at 96 °C for 15 s, annealing at 55 °C for 60 s and extension at 72 °C for 30 s. PCR was finished by final extension at 72 °C for 60 s. Amplicons (308 bp) were analyzed on 1.5% agarose gel, stained with ethidium bromide and visualized under UV light.

For isolates possessing *mcr*-1 gene, MICs were determined with RUO E-test colistin CO 256 according to the manufacturer’s guidelines (bioMérieux Deutschland GmbH). Briefly, an overnight bacterial suspension in Mueller–Hinton broth was adjusted to a density of McFarland 0.5 evenly streaked on Mueller–Hinton agar plates to ensure uniform growth. Once the agar surface was dry, an E-test^®^ colistin strip (concentration range from 0.016 to 256 μg/ml) was applied to the plate with sterile forceps. The MIC was determined after aerobic incubation for 20 h at 37 °C as the point, where inhibition of bacterial growth intersected the E-test strip. According to clinical breakpoints given by EUCAST, an isolate was defined as resistant to colistin when the MIC value was > 2 µg/ml [48].

## Results

### Isolation and identification of *Enterobacteriaceae*

Out of 576 samples, 72 *Enterobacteriaceae* isolates were identified by MALDI-TOF MS. The isolates were classified as 63 *E. coli* (87.5%), 5 *Enterobacter cloacae* (6.9%), 2 *K. pneumoniae* (2.8%) and 2 *Citrobacter* spp. (2.8%).

Seven out of 63 *E. coli* isolates could not be re-cultivated for testing of antibiotic resistance after applying MALDI-TOF MS (11.1%), while DNA was extracted from preserving solution for further identification.

### Antimicrobial susceptibility testing

The results of phenotypic antibiotic susceptibility testing of 56 re-cultivated *E. coli* isolates were given in Table 2. *E. coli* isolates showed high resistance rates to penicillin, erythromycin and rifampicin with 98.2, 96.4 and 96.4%, respectively. Resistance rates to other tested antibiotics were between 10.7% for amikacin and 64.3% for trimethoprim/sulfamethoxazole. Only one *E. coli* isolate (1.8%) was resistant to imipenem (Tables 2 and 6). Fifty-five out of 56 *E. coli* isolates (98.2%) were resistant to antibiotics of at least three different classes of antimicrobial agents and thus they were defined as multidrug resistant isolates (Table 2).

The antimicrobial susceptibility profiles for other species of *Enterobacteriaceae* were presented in Table 3. All 5 *Enterobacter cloacae* isolates were resistant to penicillin, erythromycin and rifampicin. Two *Citrobacter* spp. isolates were resistant to penicillin, rifampicin, streptomycin and ceftazidime. Two *K. pneumoniae* strains were sensitive to amikacin and imipenem but resistant to the rest of the antibiotics tested.

### Genoserotyping of *E. coli* isolates using microarray analysis

Three out of 63 *E. coli* isolates (4.8%) were determined as O91 and O15; in all other cases O type determination failed. H antigen types were identified for all isolates. Seventeen different types of H antigens (H1, H2, H4, H5, H6, H7, H8, H10, H11, H16, H19, H21, H26, H28, H32, H34 and H51) were detected. H21 (14 isolates), H28 (10 isolates) and H51 (8 isolates) are being the most common types.

### Detection of antibiotic resistance determinants in *E. coli* by microarray analysis

Several resistance genes were identified in 15 phenotypically resistant *E. coli* using microarray analysis (Table 4). The isolates were originated from four districts located in four provinces, namely Dakahliya (n = 7), Damietta (n = 3), Gharbiya (n = 3) and Kafr El-Sheikh (n = 4). Frequently identified resistance genes were *aad*A1 associated with resistance to aminoglycosides (n = 12), *sul*2 responsible for sulphonamide resistance (n = 10) and *flo*R connected with resistance to chloramphenicol (n = 10).

**Table 4:** Genoserotypes and resistance profiles of *Escherichia coli* isolates possessed resistance genes from different farms in four districts in Egypt

District	Isolate	O-type	H-type	Virulence genes	Resistance genes	Phenotypic resistance	ESBL
Dakahliya (n = 7)	16CS0049	–	5	*hem*L	*qnr*S, *tet*A, *bla*_TEM_	TET, RAM, ERY, PEN	
	16CS0070	–	51	*ipf*A*, cma, hem*L*, intl*1*, iro*N*, iss*	*sul*3, *cml*A1, *flo*R, *aad*A1, *aph*A, *str*B, *mph*A, *mrx*, *bla*_TEM_	CIP, TET, CMP, RAM, GEN, STR, ERY, PEN	
	16CS0071	–	21	*ipf*A*, cma, intl*1*, iro*N*, iss*	*sul*1, *sul*2, *dfr*A12, *tet*A, *flo*R, *aad*A1, *mph*A, *mrx*, *bla*_CMY_, *bla*_TEM_	CIP, LEV, T/S, TET, CMP, RAM, GEN, STR, ERY, PEN, AMP, CAZ	+
	16CS0078	–	4	*hem*L*, intl*1*, iss*	*sul*2, *dfr*A14, *tet*A, *str*B	T/S, TET, RAM, STR, ERY, PEN	
	16CS0740	–	26	*cif, esp*A*, esp*F*_O103H2, esp*J*, nle*A*, nle*B*, O157:H7, tcc*P*, ast*A*, hem*L*, intl*1*, tir*, *eae*	*sul*1, *sul*2, *sul*3, *dfr*A14, *tet*A, *cml*A1, *flo*R, *arr*, *aad*A1, *aad*B, *ant*2, *aph*A, *str*B, *mph*A, *mrx*, *bla*_OXA-7_	CIP, T/S, TET, CMP, RAM, GEN, STR, ERY, PEN, AMP	+
	16CS0744	–	51	*cma, hem*L*, intl*1*, iro*N	*sul*3, *dfr*A1, *dfr*A14, *tet*A, *cml*A1, *aad*A1, *aph*A, *mph*A, *mrx*, *bla*_CMY_, *bla*_TEM_	CIP, T/S, TET, RAM, GEN, STR, ERY, PEN, AMP, CAZ	
	16CS0772	–	51	*ipf*A*, mch*F*, hem*L*, intl*1*, iro*N*, iss*	*qnr*S, *sul*2, *dfr*A17, *tet*B, *cat*A1, *flo*R, *aad*A1, *aad*A4, *str*B, *bla*_TEM_, *bla*_CTX-M9_	CIP, T/S, TET, CMP, RAM, GEN, STR, ERY, PEN, AMP, CAZ	+
Damietta (n = 3)	16CS0069	–	51	*cma, intl*1*, iro*N*, iss*	*sul*3, *dfr*A12, *cml*A1, *aad*A1, *mph*A, *mrx*	CIP, LEV, T/S, CMP, RAM, STR, ERY, PEN, AMP	
	16CS0743	–	51	*mch*F*, hem*L*, intl*1*, iro*N*, iss*	*sul*2, *dfr*A17, *tet*B, *cat*A1, *aad*A4, *bla*_TEM_	CIP, T/S, TET, CMP, RAM, STR, ERY, PEN	
	16CS0752	–	6	*iha, prf*B*, sat, hem*L*, intl*1	*sul*1, *sul*3, *dfr*A12, *cml*A1, *flo*R, *aac*6, *aac*6 lb, *aad*A1, *aad*A2, *aph*A, *mph*A, *mrx*, *bla*_CTX-M1_/*bla*_CTX-M15_, *bla*_OXA-1_, *bla*_TEM_	not determined	
Gharbiya (n = 3)	16CS0747	–	32	*hem*L*, intl*1*, ire*A	*qnr*S, *sul*3, *tet*A, *cml*A1, *flo*R, *arr*, *aad*A1, *bla*_OXA-7_, *bla*_SHV_, *bla*_TEM_	T/S, TET, CMP, RAM, GEN, STR, ERY, PEN AMP, CAZ	+
	16CS0761	–	51	*hem*L*, intl*1*, iro*N*, iss*	*qnr*S, *sul*2, *dfr*A1, *dfr*A17, *tet*A, *tet*C, *cat*A1, *flo*R, *aad*A1, *aad*A4, *aph*A, *str*B, *ere*A, *bla*_TEM_, *bla*_LAP-1_	CIP, LEV, T/S, TET, CMP, RAM, GEN, STR, ERY, PEN, AMP	+
	16CS0762	–	51	*ipf*A*, mch*F*, hem*L*, intl*1*, intl*2*, iro*N*, iss*	*sul*2, *dfr*A1, *dfr*A17, *tet*A, *tet*B, *tet*C, *cat*A1, *flo*R, *aad*A1, *aad*A4, *aph*A, *str*B, *ere*A, *bla*_TEM_	not determined	
Kafr El-sheikh (n = 4)	16CS0067	15	1	*prf*B*, sen*B*, hem*L*, iss*	*qnr*S, *sul*1, *sul*2, *dfr*A7, *dfr*A17, *dfr*A19, *tet*A, *aad*A4, *str*A, *str*B, *mph*A, *mrx*, *bla*_TEM_	T/S, TET, RAM, GEN, STR, ERY, PEN	
	16CS0075	–	51	*ipf*A*, cma, hem*L*, intl*1*, iro*N*, iss*	*sul*3, *cml*A1, *flo*R, *aad*A1, *aph*A, *mph*A, *bla*_TEM_	CIP, TET, CMP, RAM, GEN, STR, ERY, PEN	
	16CS0755	–	10	*prf*B*, cme, intel*1*, iro*N*, iss*	*sul*2, *sul*3, *dfr*A1, *cml*A1, *flo*R, *aad*A1, *aph*A, *ere*A, *bla*_TEM_	CIP, LEV, T/S, TET, CMP, RAM, GEN, STR, ERY, PEN, AMP, CAZ	+
	16CS0774	–	1	*ipf*A*, prf*B*, tsh, mch*F*, hem*L*, ire*A*, iro*N*, iss*	*qnr*B, *sul*1, *sul*2, *dfr*A7, *dfr*A17, *dfr*A19, *tet*B, *aph*A, *bla*_DHA-1_	T/S, TET, RAM, STR, ERY, PEN, AMP, CAZ	+

In this study, five *E. coli* isolates harboured *qnr*S gene while one isolate possessed *qnr*B gene associated with quinolone resistance. In two phenotypically ciprofloxacin-resistant *E. coli*, *qnr*S gene was detected (Table 4).

The *sul* and *dfr*A genes associated with sulphonamides/trimethoprim resistance were detected in 16 and 13 *E. coli* isolates, respectively (Table 4). Meanwhile, *sul*3 gene corresponding to sulphonamide resistance was amplified in two susceptible *E. coli* to sulphonamide/trimethoprim.

Eleven *E. coli* phenotypically resistant to tetracycline were harboured one or more *tet* genes (*tet*A, *tet*B or *tet*C) (Table 4). Chloramphenicol resistance-associated genes *cat*A1, *cat*B3, *cml*A1 and *flo*R were detected in 13 *E. coli* isolates. Out of 13 chloramphenicol resistant isolates, 10 isolates harboured one or more resistance genes. The *cml*A1 gene was detected once in an *E. coli* strain that was phenotypically susceptible to chloramphenicol.

Ten different genes (*aac*6, *aac*6Ib, *aad*A1, *aad*A2, *aad*A4, *aad*B, *ant*2, *aph*A, *str*A and *str*B) associated with aminoglycoside resistance were detected in 14 out of phenotypically tested *E. coli* isolates (Table 4). All isolates harbouring at least one of described genes were phenotypically resistant to streptomycin, but four of them were susceptible to gentamicin (Tables 3, 4). All isolates with aminoglycoside resistance-associated genes were susceptible to amikacin.

Genes associated with macrolide resistance (*ere*A, *mph*A, *mrx*) were identified in 9 phenotypically resistant *E. coli* to erythromycin. The rifampicin resistance gene *arr* was identified in only 2 phenotypically rifampicin resistant isolates.

Fifteen out of 63 *E. coli* isolates (23.8%) harboured one or more ESBL, narrow-spectrum β-lactamase (NSBL) or AmpC β-lactamase genes. The gene *bla*_TEM_ was found in 13 DNAs of *E. coli* isolates (20.6%), *bla*_CMY_ and *bla*_OXA-7_ were detected in 2 samples each (3.2%) and *bla*_SHV_, *bla*_OXA-1_, *bla*_CTX-M1/15_ and *bla*_DHA-1_ were found in one isolate (1.6%).

The correlation between the genotypic and phenotypic antimicrobial resistance of *E. coli* was demonstrated in Table 4.

Thirteen out of 15 isolates harbouring *bla* genes were analyzed using the VITEK-2 (Table 5). Two samples could not be tested, as they could not be re-cultivated. All isolates possessing beta-lactam resistance genes were resistant to piperacillin, while one isolate was susceptible to moxifloxacin. All isolates were susceptible to fosfomycin. The narrow-spectrum beta-lactamase gene *bla*_OXA-1_ was detected once in one ESBL isolates originated from poultry farm in Damietta.

**Table 5:** Results of antimicrobial resistance testing using VITEK-2 system

Isolate	PIP	TZP	CTX	CAZ	FEB	ATM	IMP	MEM	AMK	GEN	TOB	CIP	MXF	TGC	FOS	CT	T/S	
16CS0049	R	I	S	S	S	S	S	S	S	S	S	I	R	S	S	S	S	No ESBL
16CS0070	R	I	S	S	S	S	S	S	S	R	R	R	R	S	S	S	S	No ESBL
16CS0071	R	I	R	R	I	R	S	S	S	R	R	R	R	S	S	S	R	ESBL
16CS0075	R	I	S	S	S	S	S	S	S	R	R	R	R	S	S	R	S	No ESBL
16CS0743	R	I	S	S	S	S	S	S	S	S	S	R	R	S	S	S	R	No ESBL
16CS0744	R	I	R	R	I	R	S	S	S	R	R	R	R	S	S	R	R	ESBL
16CS0747	R	R	R	R	I	R	I	I	S	R	R	I	R	S	S	S	S	ESBL/Carba
16CS0761	R	I	R	I	I	I	S	S	S	R	R	R	R	S	S	S	R	ESBL
16CS0772	R	R	R	R	I	R	S	S	S	R	R	R	R	S	S	S	R	ESBL
16CS0774	R	I	I	R	I	R	S	S	S	S	S	S	S	S	S	S	R	ESBL
16CS0067	R	I	S	S	S	S	S	S	I	R	R	S	R	S	S	S	R	No ESBL
16CS0740	R	I	S	S	S	S	S	S	S	R	R	R	R	S	S	S	R	No ESBL
16CS0755	R	I	R	R	I	R	S	S	S	R	R	R	R	I	S	S	R	ESBL

*PIP* piperacillin, *TZP* piperacillin/tazobactam, *CTX* cefotaxime, *CAZ* ceftazidime, *FEB* cefepime, *ATM* aztreonam, *IMP* imipenem, *MEM* meropenem, *AMK* amikacin, *GEN* gentamicin, *TOB* tobramycin, *CIP* ciprofloxacin, *MXF* moxifloxacin, *TGC* tigecycline, *FOS* fosfomycin, *CT* colistin, *T/S* trimethoprim/sulfamethoxazole

### Genotypic and phenotypic identification of resistance to colistin

Plasmid-mediated colistin resistance gene *mcr*-1 was detected in 5 out of 63 *E. coli* (8.0%) using a PCR assay. All of them were phenotypically confirmed as resistant to colistin using E-test (Table 6). All colistin resistant *E. coli* isolates were phenotypically resistant to rifampicin, penicillin and erythromycin but were susceptible to carbapenems. The colistin-resistant isolates originated from different poultry flocks in Dakahliya, Kafr El-Sheikh, Damietta and Gharbiya (Table 6).

**Table 6:** Characterization of colistin-resistant and carbapenemase-producing *E. coli* isolates

Isolate	Governorate	Farm	O-antigen	H-antigen	Resistance genes	Virulence genes	Phenotypic resistance	*mcr*-1	MIC value	ESBL	Carbapenemase
16CS0744	Dakahliya	9	–	51	*sul*3, *dfr*A1, *dfr*A14, *tet*A, *cml*A1, *aad*A1, *aph*A, *mph*A, *mrx*, *bla*_CMY_, *bla*_TEM_	*cma, hem*L*, int*l1*, iro*N	CIP, T/S, TET, RAM, GEN, STR, ERY, PEN, AMP, CAZ	+	≥ 4	Positive	Negative
16CS0078	Dakahliya	16/2	–	4	*sul*2, *dfr*A14, *tet*A, *str*B	*hem*L*, intl*1*, iss*	T/S, TET, RAM, STR, ERY, PEN	+	≥ 6	nd by VITEK	nd by VITEK
16CS0075	Kafr El-Sheikh	9	–	51	*sul*3, *cml*A1, *flo*R, *aad*A1, *aph*A, *mph*A, *bla*_TEM_	*cma, hem*L*, intl*1*, iro*N*, iss*	CIP, TET, CMP, RAM, GEN, STR, ERY, PEN	+	≥ 32	Negative	Negative
16CS0775	Damietta	4	–	4	–	*pic*, *vat*, *cma*, *hly*E, *mch*F, *hem*L, *ire*A, *iro*N, *iss*	TET, RAM, ERY, PEN	+	≥ 6	nd by VITEK	nd by VITEK
16CS0036	Damietta	6	–	21	–	*hem*L	RAM, ERY, PEN, AMC	+	≥ 4	nd by VITEK	nd by VITEK
16CS0747	Gharbiya	4	–	32	*qnr*S, *sul*3, *tet*A, *cml*A1, *flo*R, *arr*, *aad*A1, *bla*_OXA-7_, *bla*_SHV_, *bla*_TEM_	*hem*L*, intl*1*, ire*A	T/S, TET, CMP, RAM, GEN, STR, ERY, PEN AMP, CAZ	−	nd	Positive	Positive

### Microarray analysis concerning virulence-associated genes

The virulence genes detected by microarray were differently distributed all over the isolated *E. coli*. One isolate 16CS0740 isolated from poultry farm in Dakahliya harboured 7 genes of virulence-associated secretion system: *cif, esp*A, *esp*F*_*O103H2, *esp*J, *nle*A, *nle*B O157:H7 and *tcc*P.

*eae* and *iha* genes, involved in adhesion, were identified in 16CS0740 and 16CS0752, repectively. Two isolates harboured serine protease autotransporter genes. 16CS0774 carried *tsh* while 16CS0775 had *pic* and *vat* genes.

Several toxin genes were detected in 13 *E. coli* isolates including *ast*A, *cma*, *hly*E, *mch*F, *sat* and *sen*B. Each of these isolates carried only one toxin gene except 16CS0775 which harboured *mch*F, *hly*E and *cma*. Nineteen out of 63 *E. coli* isolates (30.2%) harboured *lpf*A and 3 others carried *prf*B fimbrae virulence gene. Miscellaneous genes encoding virulence factors as *hem*L, *int*I1, *ire*A, *iro*N, *iss* and *tir* genes were identified in 45 (71.4%), 10 (15.9%), 3 (4.8%), 9 (14.3%), 36 (57.1%) and 1 (1.6%) of 63 isolates, respectively.

The distribution of virulence-associated genes in *E. coli* isolates possessed antimicrobial resistance-associated genes was demonstrated in Table 4.

## Discussion

*Escherichia coli* is a commensal pathogen of the intestinal tract of young and adult poultry [49]. Among healthy chickens, 10 to 15% of intestinal coliform bacteria may belong to potentially pathogenic serotypes of *E. coli* [50].

The identification of bacterial foodborne pathogens of zoonotic significance using rapid, accurate and reliable tools such as MALDI-TOF MS is mandatory for public health surveillance [44, 51].

In 2016, 576 cloacal swabs were collected from 48 poultry farms located in 5 governorates in northern Egypt. The samples were screened for multidrug resistant bacteria and investigated for the antimicrobial resistance of *E. coli*. Seven out of 56 *E. coli* isolates (12.5%) were producing ESBLs. To analyze the underlying molecular antimicrobial resistance mechanism, all *E. coli* isolates were genotyped using the multiplex microarray technique.

The results of this study were in accordance with previous reports which demonstrated a high prevalence of *E. coli* in poultry farms and their environment in Egypt [34, 52–54].

In previous studies on broiler chickens in Egypt, high phenotypic resistance rates were found to penicillin, rifampicin, erythromycin, trimethoprim/sulphamethoxazole, streptomycin and tetracycline [53]. Antimicrobial resistance rates in this study for amoxicillin (26.8%), gentamicin (19.6%) and imipenem (1.8%) were lower than those of *E. coli* isolates from poultry reported in Egypt [53], in China [55], in United States [56], in Korea [57], in United Kingdom [58], in Australia [59] and in Portugal [60]. In the present investigation, the most striking finding was that *E. coli* isolates showed a low resistance rate to fluoroquinolones (ciprofloxacin (21.4%) and levofloxacin (14.3%)). Cephalosporins are the first-line antimicrobials for treating human bacterial infections [61]. In addition, a considerable resistance to ceftazidime was detected among *E. coli* isolates from healthy broilers (41.1%).

In this study, one carbapenem-resistant isolate (1.8%) was found within all *E. coli* isolates. A higher rate was determined with retail chicken meat, 11.3% carbapenemase-producing *Enterobacteriaceae* including *E. coli* in Egypt in a previous study [62].

Few reports discussed prevalence of ESBL-producing *E. coli* isolated from healthy birds in Egypt. Here, ESBL and/or AmpC β-lactamase-producing isolates were detected in seven out of tested 56 *E. coli* (12.5%) isolated from healthy broilers. Two ESBL-producing strains were isolated from one farm in Gharbiya governorate (Farm 4) while five other isolates could be recovered from different farms in Dakahliya and Kafr El-Sheikh governorates. Two of the ESBL-producing isolates 16CS0740 and 16CS0747 from Dakahliya and Gharbiya, respectively were additionally carrying *bla*_OXA-7_ gene characteristic for β-lactamase-producing bacteria (Table 4).

In a previous study in 2017, only 6% ESBL-producing *E. coli* were detected in colibacillosis diseased poultry in four different Egyptian governorates [63]. In contrast, ESBL/AmpC β-lactamase-producing *E. coli* were found in all 50 investigated Dutch broiler farms [64]. In Sweden 34.0% of broilers carried ESBL/AmpC β-lactamase-producing *E. coli* in their guts [65]. In Malaysia 48.8% of isolates which were recovered from retail poultry meat markets were ESBL-AmpC positive [66].

The prevalence of ESBLs has been found to be variable worldwide with Asian countries having the highest rates [67].

In this study, the most prevalent resistance gene was *bla*_TEM_, which was identified in 85.7% of ESBL and AmpC β-lactamase-producing isolates. *bla*_CMY-2_ was found in 2 of ESBL and AmpC β-lactamase-producing isolates. *bla*_OXA-7_ was found in 2 of ESBL producing isolates. While *bla*_CTX-M9_, *bla*_CTX-M1-15_, *bla*_OXA-1_, *bla*_DHA-1_, *bla*_LAP-1_ and *bla*_SHV_ were identified only in one ESBL-producing *E. coli* isolate.

The resistance-associated genes *bla*_TEM_, *bla*_SHV_, and *bla*_CMY_ were previously reported in *Enterobacteriaceae* isolated from septicaemic broilers [68] and from humans [69, 70] in Egypt.

In this study, *bla*_TEM_ resistance gene was detected in 20.6% of *E. coli* isolates. This result was in accordance with previous reports in China [71, 72]. *bla*_CMY_ was detected in 3.5% of 56 *E. coli* isolates while the prevalence of *bla*_CMY-2_ amongst *E. coli* isolates from broilers in Japan was 69.5% [73]. In Belgium, 49.0% of ceftiofur-resistant *E. coli* isolates derived from five broiler farms carried *bla*_CMY-2_ [74]. Moreover, 12.1% of avian pathogenic *E. coli* strains and 9.5% of strains recovered from meat were found positive as carriers of *bla*_CTX-M_ in Palestine [75].

*qnr*B and *qnr*S genes associated with quinolone resistance were detected in one and five isolates, respectively, which is lower than described previously in *E. coli* isolated from chickens in China [72, 76]. On the other hand, *qnr*A, *qnr*B, and *qnr*S genes were detected in 0.75, 3.9 and 5.1%, respectively of *E. coli* from chicken samples in China [77].

Many studies found similarities between virulence-associated genes in human and avian *E. coli* isolates including *iss*, *fli*C, *iha* and *ire*A genes [78].

In a previous study, the virulence genes *iro*N, *omp*T, *iss*, *iut*A, and *hly*F were detected in 80.2% of isolated *E. coli* [68]. In this study, only 9 (16.7%) of the 56 *E. coli* isolates carried 2 genes (*iro*N, *iss*) together characteristic for avian pathogenic *E. coli*.

The *mcr*-1 gene is now reported all over the world in *Enterobacteriaceae* from animals, food and humans [79]. In 2015, first time *mcr*-1 gene was detected in livestock and raw meat samples in addition to human beings in China [27]. In this study, five *E. coli* isolates (8.9%) were phenotypically resistant to colistin and harboured *mcr*-1 gene associated with colistin resistance. This result was higher than reported in *E. coli* isolates from pigs, poultry and turkey in France with 0.5, 1.8 and 5.9%, respectively [80] and 5.6% of *E. coli* isolates from broilers in Germany [81], while it was lower than in *E. coli* isolates from poultry in China [27].

In previous studies conducted in China and Austria, the majority of phenotypically colistin-resistant *E. coli* isolates carried the *mcr*-1 gene [82, 83].

## Conclusion

To the best of our knowledge, this study is the first report discussing the antibiotic susceptibility profiles of *Enterobacteriaceae* and ESBL-producing *E. coli* isolated from healthy broilers in the Nile Delta in Egypt. The emergence of colistin-resistant *E. coli* isolates in poultry is of public health significance and considered as potential source of transmission of plasmid-mediated *mcr*-1 to humans. It was shown that molecular biological methods such as microarray investigation are reliable and fast tools for detection of geno-serotypes, resistance- and virulence-associated determinants.

The results reinforce the need to develop surveillance strategies and to implement specific control procedures to reduce the use of antibiotics and subsequently the development of antimicrobial resistance by over-/misuse of antibiotic agents.
